# Chemical cues of an invasive turtle reduce development time and size at metamorphosis in the common frog

**DOI:** 10.1038/s41598-020-64899-0

**Published:** 2020-05-14

**Authors:** M. Vodrážková, I. Šetlíková, M. Berec

**Affiliations:** Faculty of Agriculture, University of South Bohemia in České Budějovice, Studentská 1668, České Budějovice, Czech Republic

**Keywords:** Ecology, Zoology

## Abstract

In aquatic systems, chemical cues are one of the major sources of information through which animals can assess local predation risk. Non-native red-eared sliders (*Trachemys scripta elegans*) have the potential to disrupt aquatic ecosystems in Central Europe because of their superior competitive abilities and omnivorous diets. In this study, we examined whether continuous predator-borne cues are tied to changes in the developmental rates, growth rates and sizes at metamorphosis of common frog tadpoles (*Rana temporaria*). Our results show rather rarely documented types of amphibian prey responses to caged predators. The presence of turtles shortened the time at metamorphosis of tadpoles from 110 ± 11.7 days to 93 ± 13.0 days (mean ± S.D.). The first metamorphosed individuals were recorded on the 65^th^ day and on the 80^th^ day from hatching in the predator treatment and in the control group, respectively. The froglets were significantly smaller (12.8 ± 0.99 mm) in the presence of the predator than in the control treatment (15.2 ± 1.27 mm). The growth rate trajectories were similar between the predator treatment and the control. Thus, predator-induced tadpole defences were evident in higher developmental rates and smaller sizes at metamorphosis without significant changes in growth.

## Introduction

Alien species are recognized as important drivers of global environmental change^1,2^. Although only a fraction of alien species become invasive^3^, the massive increase in the rate of biological invasions due to rapid globalization of trade is reflected in the corresponding increase in research into the impacts of invasive alien species^4^. In addition to damage to human health and economies^5^, alien species can have a significant impact on the functioning of parts of communities, entire communities or local ecosystems^6,7^. Alien species can interfere with the life histories of autochthonous fauna in many ways, including through predation, competition, transmission of new diseases, immune system compromisation, or hybridization^8,9^.

Many amphibian species are unable to respond to non-native predators^10^ however, there is a plethora of studies that documented measurable behavioural, physiological or morphological reactions. The ability to detect and recognize potential predators is an important component of the anti-predatory behaviour of amphibians in all their developmental stages^11^. Prey responses to the threat of predation are multi-faceted and include increased refuge use^12,13^, increased distance from the threat^14^, reduction of activity^15–18^, shortening of feeding time^19,20^, change in development time^21–23^, change in growth rate^24^, reduced size at metamorphosis^25^, changes in body shape^26–28^, changes in colour^29^ and inedibility^13,30^.

Werner^31^ predicted that the exact point at which metamorphosis is favoured depends on the equilibrium of size-specific growth and mortality rates in aquatic and terrestrial habitats. A new predator in the aquatic environment can fundamentally impairs the habitat for amphibian larvae. A rapid growth theoretically provides the best protection against the costs of predation risk. Exposure to predators can also alter rates of development and progress toward metamorphosis. Surprisingly, the majority of studies demonstrated that exposure to predators resulted in longer or the same time of aquatic phase duration and larger or the same size at metamorphosis^32^.

In their native range, red-eared sliders (*Trachemys scripta elegans*) are opportunistic omnivores. All turtle size classes consume significant proportion of animal matter, which makes them potentially dangerous to native species^33,34^. This turtle species is currently the world’s most widespread freshwater turtle, because it has been widely introduced, both purposely or as the side effect of the pet trade, all over the world with the exception of Antarctica^35,36^. Although turtles were not mentioned among the non-native predators of amphibians^8^, our previous research^18^ revealed that tadpoles of common frogs (*Rana temporaria*) recognize red-eared sliders as potential predators. In that study^18^, we documented that in the presence of turtle kairomones, tadpoles significantly reduce their movement activity (by almost 50%) and modify their swimming paths into more zigzagged trajectories. Because this type of antipredator behaviour is expected to convey costs in terms of lost foraging opportunities, we examined whether naïve common frog tadpoles respond adaptively to the presence of turtle kairomones^37^. Specifically, we asked if the presence of turtle cues accelerate the developmental or growth rate in tadpoles of common frog in laboratory conditions as predicted by theoretical models^31^ or decelerate as was often experimentally documented^32^. Secondly, we addressed the question of whether tadpoles respond to the presence of turtle cues by shifting body size at metamorphosis.

## Materials and methods

In the experiment, two adult red-eared sliders (carapax length: 18 cm and 21 cm) and the tadpoles of common frogs were used. Six clutches of common frog eggs were collected in pools around Holubov, South Bohemia, Czech Republic (48.9078633N, 14.3485608E) on 15-Apr-2017. Neither the eggs nor their parents came into contact with red-eared sliders at this locality. Clutches were placed in a glass tank of 220 L volume with tap water and a pump filter in a temperature-controlled laboratory room (20 ± 1 °C) for fourteen days. The pump filter was rinsed twice a week. The turtles were fed three times a week with chicken meat and ReptoMin Tetra turtle gammarus. The tadpoles were fed daily with TetraMin aquarium flakes for ornamental fish. The light source was a fluorescent tube (2 × 36 W) with a light regime of 12 h/12 h.

Four glass tanks of 220 L volume (size: 100 × 40 × 55 cm) and 6 cm water depth were used for the experiment. A Claro 300 filter pump (300 L.h^−1^) was installed in each glass tank. To prevent physical, but not chemical, contact between turtle and tadpoles, a glass barrier was placed inside each glass tank with a 6 cm gap at both ends so that water could flow freely throughout the tank. On the other side of the barriers, 25 individual perforated opaque boxes (8 × 8 cm) with holes 2 mm in diameter were glued to the bottoms of the glass tanks (5 rows with 5 boxes in each row). Tadpoles of the same size (16.6 ± 0.16 mm) at stages 26 and 27 according to Gosner^38^ were stocked individually in each box, and turtles were placed in two of the glass tanks prior to the experiment. During the experiment, the same feeding regime as that in the preparation phase was used. The tadpoles were photographed under a stereomicroscope (Olympus SZX 7) and measured using the QuickPHOTO MICRO 3.1 program every 14 days for 112 days. Tadpoles who died prior to metamorphosis (2%; one individual in each control glass tank) were eliminated from the experiment.

Linear regression between size and time was used to obtain slopes of individual growth trajectories (for all cases, R^2^ > 0.820, *P* < 0.05). A hierarchical ANOVA with predator presence/absence as a fixed factor and glass tank identity (1–4) as a random factor nested in predator was used to test the differences in development time, final size of froglets at metamorphosis and slopes of individual growth. The normality and the homogeneity of the variances were checked using the Shapiro-Wilk test and the Bartlett test, respectively. All data analyses were performed in Statistica 13.

All methods were carried out in accordance with relevant guidelines and regulations. All experimental protocols were approved by the Czech Ministry of Agriculture, Department of Animal Welfare according to article No. 15, section 2 of the act registered under number 9103/2009-17210.

## Results

**Figure 1:**
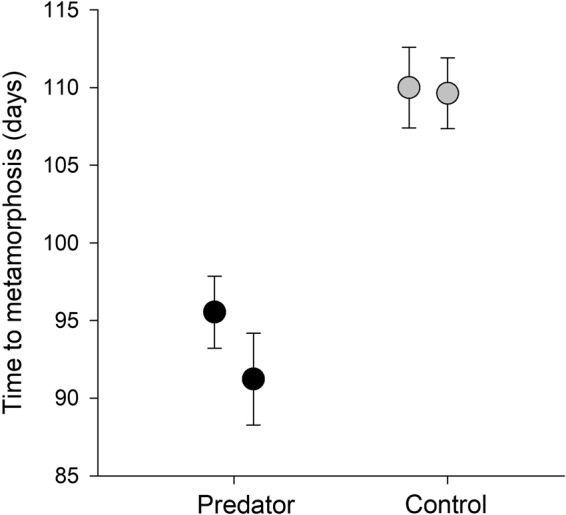
Time to metamorphosis of *Rana temporaria* tadpoles in the presence of predator (*Trachemys scripta elegans*) and those in the control group (mean ± S.E.). The average time to metamorphosis were longer when tadpoles were exposed to the predator. Each point represents one glass tank.

The time to metamorphosis differed significantly between the treatment with predator presence and control treatment (F_1,2_  = 56.99, *P*  =  0.017). The average time to metamorphosis were 93 ± 13.0 days and 110 ± 11.7 days (mean ± S.D.) in the group where tadpoles were exposed to the predator and in the control group, respectively (Fig. 1). In the predator treatment group, the first metamorphosed individuals were recorded on the 65^th^ day after hatching. The tadpoles in the control group achieved metamorphosis for the first time on the 80^th^ day. The impact of the individual glass tanks was not proven for time to metamorphosis (F_2,94_  = 0.74, *P*  = 0.481).

**Figure 2:**
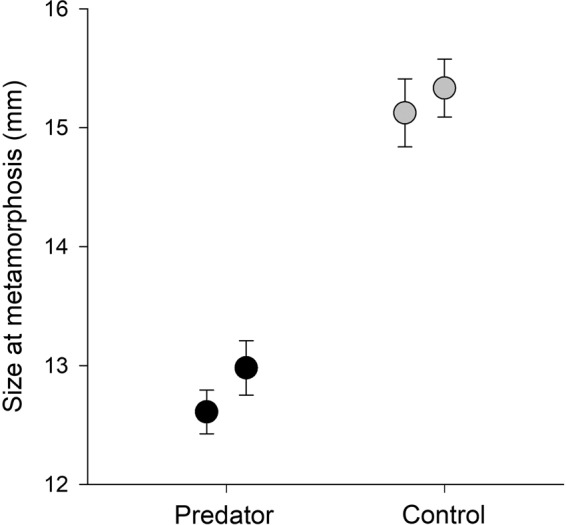
Size at metamorphosis of *Rana temporaria* froglets in the presence of predator (*Trachemys scripta elegans*) and those in the control group (mean ± S.E.). The average size at metamorphosis were larger when tadpoles were exposed to the predator. Each point represents one glass tank.

**Figure 3:**
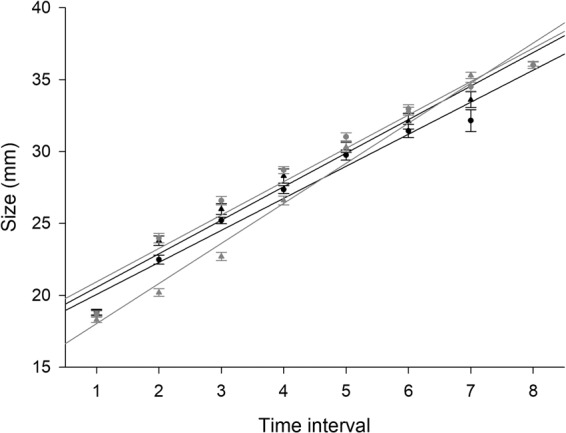
Growth trajectories of tadpoles of *Rana temporaria* in the presence of the predator (*Trachemys scripta elegans*) (black) and in the control group (grey). Slopes of growth trajectories were similar between the treatments but differed among the glass tanks. Each time interval corresponds to 14 days from the start of the experiment (mean ± S.E.). Each line represents one glass tank in the presence of the predator and in the control group.

Similarly, we found a significant difference in the size at metamorphosis of froglets raised with the predator and without it (F_1,2_  = 130.76, *P*  =  0.008). The froglets were significantly smaller (12.8 ± 0.99 mm) in the presence of the predator than those in the control group (15.2 ± 1.27 mm) (mean ± S.D.) (Fig. 2). The impact of the individual glass tanks was not proven for size at metamorphosis (F_2,94_  = 0.81, *P*  = 0.447). Slopes of individual growth trajectories did not differ between the treatments (F_1,2_  = 0.51, *P*  =  0.549), although individual glass tanks differed significantly (F_2,94_  = 8.18, *P*  = 0.001), specifically one control tank differed from the others (Fig. 3).

## Discussion

This study clearly shows that common frog tadpoles recognize the presence of alien red-eared sliders through water-borne chemical cues and react to the potential danger by shaping their developmental trajectories through a physiological life-history shift, in which final size was traded off against development rate. Our previous study^18^ of common frog tadpoles from the same population showed that naïve tadpoles without any previous exposure or contact to red-eared sliders are able to innately face and respond to the water-borne chemical cues of the turtles by modifying their swimming behaviour. This study further corroborates these findings by finding that the presence of alien turtles also induces developmental plasticity of tadpoles. Invasive predators may often not be recognized as enemies by native prey (“prey naiveté hypothesis”)^39–41^. The red-eared slider was specifically used as a potential alien predator of tadpoles in only two studies what we are aware of, in which an ability to recognize non-native turtles as a predator was either demonstrated^18^ or not^42^. If alien predator is innately recognized as an enemy, this ability is attributed to the cue similarities between alien and native predators or to the generality of the cues used by naïve prey to assess risk predation^42,43^. However, this might not be the case in our study, because the tadpoles in our experiment originated from a population that lives in a geographic area with a complete absence of turtles. An alternative explanation, although much less probable^44–46^, would assume the similarity of the cues between the red-eared slider and some of the non-turtle native predators.

The flexibility of the timing of and size at metamorphosis can be adaptive, allowing amphibian larvae to respond to changes in the quality of their aquatic environment^22,47,48^ and to increase developmental success^49,50^. In this experiment, tadpoles managed to shorten the duration of the larval period by accelerating their development rate, but not growth rate, when exposed to the constant presence of turtle chemical cues. Specifically, tadpoles metamorphosed on average 17 days earlier and at smaller size (by 16% on average) in the continual presence of the turtles than in the control treatment. This is consistent with the model of amphibian metamorphosis^31,51^. However, this type of response was only rarely demonstrated in amphibians - smaller size at metamorphosis was confirmed in only 14% of the studies, and a shorter time to metamorphose in only 5% of the studies reviewed by Relyea^32^, i.e., in the northern red-legged frog, *Rana aurora*^52^, and in the common toad, *Bufo bufo*, for shorter time to metamorphose only^53^. Comparison of growth trajectories is missing in the literature, the metamorphic changes are expressed only as body size (body mass) at the time of metamorphosis and/or time to metamorphosis^32^. However, it is also evident from these data that although acceleration of growth is one of the predicted options to avoid predation pressure, this method is very rare in amphibians^52,54^. Like most other amphibians tested, tadpoles of common frog did not accelerate growth.

The question arises, why our results are consistent with the model predictions^31,51^ unlike most other studies demonstrating mostly longer time to metamorphosis at the same size in the presence of a predator^32^. Reduction of tadpoles’ activity due to predator presence itself is sufficient to induce earlier metamorphosis at the smaller size^55^ via the increase level of corticotropin-releasing hormone^56,57^. We previously documented that common frog tadpoles decrease their activity in the presence of turtle cues^18^. A red-eared slider used as a predator model expanded the spectrum of less used vertebrate predator types and is exceptional in some features among experimental predators used so far. Moreover, this turtle is by far the largest predator ever used in this type of experiment which could lead to a significantly greater amount of released kairomones. Big size difference between predator and prey clearly limited the efficiency of tadpole to avoid predator pressure by achieving larger size^58^. Turtles therefore can act as a super stimulus and the tadpoles thus clearly responded to their presence by a rapid metamorphosis. Tadpoles try to avoid predator pressure by growing faster to be too large to become prey or to try to leave the aquatic environment as soon as possible, i.e., when tadpoles reach the minimum size at which they can metamorphose^25^. In addition, Relyea^32^ suggested that developmental plasticity can be phylogenetically constrained. Thus, the ratio of published outcome possibilities may be also biased because some of the results are due to the absence of the ability to plastically change the developmental trajectory under any conditions^32^.

In conclusion, our results suggested that common frog tadpoles are, despite the absence of a common evolutionary history with red-eared sliders, innately able to discriminate predator-specific scents of the invasive turtles and to subsequently respond to the predation risk by shortening their larval period in exchange for a smaller body size, which may affect the survival and fitness of a metamorphosed individual^51,59^. Given the probability of creating stable populations within Europe only at its southern edge, the red-eared slider is considered to have a limited impact on indigenous fauna^60^. However, the ability of escaped or released individuals to survive for a long time in the suboptimal conditions in the more northern parts of Europe^35^ makes them a potential threat to frog populations in this region. It would be very appropriate to pay future attention to the study of demecology of the post-metamorphic phase of development.
